# The Long-Term and Short-Term Efficacy of Immunotherapy in Non-Small Cell Lung Cancer Patients With Brain Metastases: A Systematic Review and Meta-Analysis

**DOI:** 10.3389/fimmu.2022.875488

**Published:** 2022-05-25

**Authors:** Xianjing Chu, Lishui Niu, Gang Xiao, Haiqin Peng, Fuxing Deng, Zhiyuan Liu, Honghua Wu, Lei Yang, Zhuguilong Tan, Zhanzhan Li, Rongrong Zhou

**Affiliations:** ^1^ Department of Oncology, Xiangya Hospital, Central South University, Changsha, China; ^2^ Xiangya Lung Cancer Center, Xiangya Hospital, Central South University, Changsha, China; ^3^ National Clinical Research Center for Geriatric Disorders, Xiangya Hospital, Central South University, Changsha, China

**Keywords:** non-small cell lung cancer, brain metastases, immunotherapy, combined immunotherapy, radiotherapy

## Abstract

**Background:**

Although immunotherapy has been widely used, there is currently no research comparing immunotherapy for non-small cell lung cancer (NSCLC) patients with brain metastases (BMs). This meta-analysis addresses a gap in the comparison of immunotherapy efficacy, including immune checkpoint inhibitors (ICIs), chemotherapy (CT), radiotherapy (RT), and ICI combined CT or RT.

**Methods:**

A search of Pubmed, Cochrane, EMBASE, and ClinicalTrial.gov was conducted to identify studies which enrolled NSCLC patients with BM treated with ICIs. The outcomes consisted of intracerebral overall response rate (iORR), intracerebral disease control rate (iDCR), extracranial overall response rate (EORR), distant brain failure (DBF), local control (LC), progression-free survival (PFS), and overall survival (OS).

**Results:**

A total of 3160 participants from 46 trials were included in the final analysis. Patients treated with immunotherapy were associated with a longer PFS (0.48, 95%CI: 0.41-0.56), and a longer OS (0.64, 95%CI: 0.60-0.69) compared with immunotherapy-naive patients. In prospective studies, dual ICI combined CT and ICI combined CT achieved a better OS. The hazard ratio (HR) of dual ICI combined CT versus dual ICI was 0.61, and the HR of ICI combined CT versus ICI monotherapy was 0.58. Moreover, no statistical difference in PFS, OS, EORR, iORR, iDCR, and EDCR was found between patients with ICI monotherapy and ICI combined cranial radiotherapy. Concurrent ICI combined RT was shown to decrease the rate of DBF (OR = 0.15, 95% CI: 0.03-0.73) compared with RT after ICI. Patients treated with WBRT might have an inferior efficacy than those with SRS because the iORR of SRS was 0.75 (0.70, 0.80) and WBRT was 0. Furthermore, no obvious difference in PFS and OS was observed among the three different types of ICI, which targets PD-1, PD-L1, and CTLA-4, respectively.

**Conclusions:**

Patients treated with ICI got superior efficacy to those without ICI. Furthermore, dual ICI combined CT and ICI combined CT seemed to be optimal for NSCLC patients with BM. In terms of response and survival, concurrent administration of SRS and ICI led to better outcomes for patients with BMs than non-concurrent or non-SRS.

**Importance of the Study:**

In the new era of immunotherapy, our meta-analysis validated the importance of immunotherapy for non-small cell lung cancer (NSCLC) patients with brain metastases (BMs). By comparing the long-term and short-term impacts of various regimens, all immunotherapy treatments had superior efficacy to immunotherapy-naive. At the same time, through pairwise comparison in immunotherapy, our findings can help clinicians to make treatment decisions for NSCLC patients with BMs.

**Systematic Review Registration:**

https://www.crd.york.ac.uk/PROSPERO/display_record.php?RecordID=269621, identifier CRD42021269621.

## Highlights

1. Immunotherapy improved OS and PFS compared to immunotherapy-naive regimens.
2. Dual ICI combined CT and ICI combined CT might be the two first-line recommendations for NSCLC patients with BM.
3. Efficacy of ICI combined RT in NSCLC patients with BM depends on the specific method of RT, and the sequence of RT and ICI.

## Introduction

Lung cancer has the characteristics of high incidence, high mortality, and low detection rate (1, 2). Metastasis is considered a leading reason for lung cancer patients’ death, especially brain metastases (BMs) (3). Based on the pathological type, lung cancer consists of small cell lung cancer (SCLC) and non-small cell lung cancer (NSCLC) (4). The mean survival time of untreated NSCLC patients with BM is as short as 1-2 months (5).

Although advances have been achieved in BM patients’ treatment recently, the survival rate is still unsatisfactory, possibly because the blood-brain barrier (BBB) hinders drug entry into the brain, such as chemotherapy (CT), of which the median overall survival was only 4-8 months (6–9). Surgery, whole-brain radiation therapy (WBRT), and stereotactic radiation surgery (SRS) are often referred to as conventional local treatments for BM (10). However, WBRT and SRS have certain limitations, such as radiation neurotoxicity, cognitive deterioration, etc. (11–13). In recent years, the emergence of tyrosine kinase inhibitors (TKIs) changed the treatments for BM, particularly in those patients with positive driver genes like epidermal growth factor receptor (EGFR), anaplastic lymphoma kinase (ALK), and c-ros oncogene 1 (ROS1) (14). Compared with first- and second-generation EGFR-TKIs, third-generation EGFR-TKIs demonstrated truly higher BBB permeability and better efficacy in patients with BM (15). Still, approximately 26% of patients with BM have no driver gene mutations (16).

Therefore, the emergence of immune checkpoint inhibitors (ICIs) that target PD-1, PD-L1, or CTLA-4 offers hope to advanced NSCLC patients with negative driver genes (17). Cohen JV et al. proposed that ICIs and active T cells can penetrate BBB (18), which is necessary for ICIs to work (19). Keynote-024 established immunotherapy as a first-line treatment for advanced NSCLC patients with positive PD-L1 (20). However, for those with unknown PD-L1 expression levels, the response rate was only 17%-19% (21–23). In addition, the results of previous clinical trials also showed that immunotherapy in combination with radiotherapy (RT) or CT might improve the survival of NSCLC patients (24–26).

However, the efficacy of immunotherapy in BM remains controversial, depending on various immunotherapy regimens (27, 28). Besides, most studies included in Alencar’s analysis (29) were retrospective with limited sample size and long-term efficacy. Although Yin et al. and Vivianedid et al. did some analyses about the efficacy of BMs immunotherapy, but the number of studies they included was limited and their analyses lacked efficacy comparison between diversified immunotherapy approaches (30, 31). Therefore, we designed and conducted this meta-analysis to evaluate the efficacy of immunotherapy more comprehensively in NSCLC patients with BM.

## Materials and Methods

### Sources of Data

This meta-analysis was performed in accordance with the Preferred Reporting Items for Systematic Review and Meta-Analyses (PRISMA) statement. And it was registered at the International Prospective Register of Systematic Reviews (PROSPERO) (number: CRD42021269621). Pubmed, Cochrane, EMBASE, Web of Science, and ClinicalTrial.gov were used to search literature by entering keywords and setting constraints. We collected all qualified clinical trials before September 25, 2021. The term words are “Carcinoma, Non-Small-Cell Lung,” “Immunotherapy,” “Immune Checkpoint Inhibitors,” “Pembrolizumab,” “Nivolumab,” “Atezolizumab,” “Durvalumab,” “Cemiplimab,” “Camrelizumab,” “Sintilimab,” “Tislelizumab,” or “Toripalimab.” And this literature retrieval process was performed independently by two authors (Xianjing Chu and Lishui Niu).

### Inclusion and Exclusion Criteria

Literature titles and abstracts were screened by two authors independently, and then the results were combined to delete duplicate results. In case of disagreement, a third researcher was required. There were no language restrictions. Studies that meet the following standards are regarded as eligible studies. The inclusion standards are: 1) the participants are stage IV NSCLC patients; 2) the type of studies is randomized controlled trials or cohort studies; 3) intervention is immunotherapy; 4) outcomes included one or more of the following indexes: iORR, iDCR, EORR, OS, PFS, DBF, LC; 5) the studies are about NSCLC with BM. Studies of reviews, editorials, comments, case reports, animal trails, or letters are excluded.

### Data Extraction

Two investigators extracted data independently by browsing full text of studies. The following information was extracted: 1) authors and publication year; 2) study type; 3) median follow-up time; 4) interventions; 5) number of total participants; 6) number of participants with BM; 7) sex; 8) age; 9) smoking history; 10) the state of driving gene mutation; 11) PD-L1 expression; 12) the histological types; 13) radiotherapy history; 14) number of metastases lesions; 15) max diameter of metastases; 16) EORR; 17) iORR; 18) iDCR; 19) hazard ratio (HR) for PFS; 20) HR for OS; 21) DBF 22) LC. Two authors independently evaluated the methodological quality (risk of bias).

For non-RCT studies, Newcastle-Ottawa Scale (NOS) was used to calculate the risk of bias. In NOS, there are three assessing criteria for cohort studies including selection of cohorts (4 points), comparability of cohorts (2 points), and assessment of outcome (3 points). For case-control studies, selection (4 points), comparability (2 points), and exposure (3 points) are key criteria. A total score of 5 or above is considered high quality (32).

For RCT studies, the risk of bias and applicability concerns graph was created by using the Cochrane risk of bias tool. Version 2 of the Cochrane risk-of-bias tool divides the main bias types into five domains which are bias arising from the randomization process, bias due to deviations from intended interventions, bias due to missing outcome data, bias in measurement of the outcome, and bias in selection of the reported result. Risk-of-bias judgments within domains were then mapped to an overall judgment for the outcome. The outcomes include high risk of bias, some concerns, and low risk of bias (33).

### Outcomes Assessing

Short-term efficacy indicators and long-term indicators are extracted. The short-term indicators include progression-free survival (PFS: the time from randomization to objective tumor progression), intracerebral objective response rate (iORR), intracerebral disease control rate (iDCR), extracranial overall response rate (EORR), extracranial disease control rate (EDCR), distant brain failure (DBF), and local control (LC), while long-term indicators consist of overall survival (OS: the time from randomization to all-cause death) (34). ORR is the sum of proportion of patients getting complete intracranial response (CR: disappearance of every target lesion, and short axis of pathological lymph nodes to be within 10 mm) and partial intracranial response (PR: at least 30% decrease of target lesions’ diameters) (35, 36). DCR refers to the ratio between patients getting complete intracranial response, partial intracranial response, and stable disease (SD: either sufficient shrinkage <30% or sufficient increase <20%). Additionally, DBF is defined as the rate between the number of patients with the appearance of new BM or a stable or decreasing lesion size and the total number of BM people. Local control (LC) is defined as a stable or decreasing lesion size (37).

### Statistical Analysis

The primary outcomes in this study were iORR, iDCR, EORR, DBF, and HRs for PFS and OS. The heterogeneity within studies was assessed using the Chi-square test and I^2^ statistics. p<0.05, or I^2^>50% indicated significant heterogeneity. The random-effect model was used for later analysis in terms of significant heterogeneity, otherwise, the fixed-effect model was used. For ORR, DCR, EORR, and DBF, the odds ratios (OR) and their 95% confidence intervals (CIs) of these outcomes were estimated. The hazard ratios (HRs) and their 95%CIs were calculated for evaluating the efficacy of the following groups: ICI vs CT, ICI+CT vs CT, ICI+RT vs ICI, and ICI+RT vs RT in NSCLC patients with BM. Subgroup analysis was performed for the sequencing of ICI and radiotherapy (concurrent vs. sequential), different intracranial radiation methods (SRS vs. WBRT), and design of studies (retrospective vs. prospective). To make the results more intuitive, forest plots were created. The pair-wise network meta-analyses of different ICI regimens and ICI types in prospective studies were performed by R version 3.2.1 and the STATA 14.0, using the fixed-effects model. Publication bias was assessed using Begg and Egger tests. Sensitivity analyses were performed for evaluating the influence of each study by omitting one study each time. Other analyses were completed using Stata software version 14.0 (Corp, College Station TX, USA) and Rev Manager 5.3. p<0.05 was considered significant unless otherwise specified.

## Results

### Search Strategy of Study Selection

**Figure 1:**
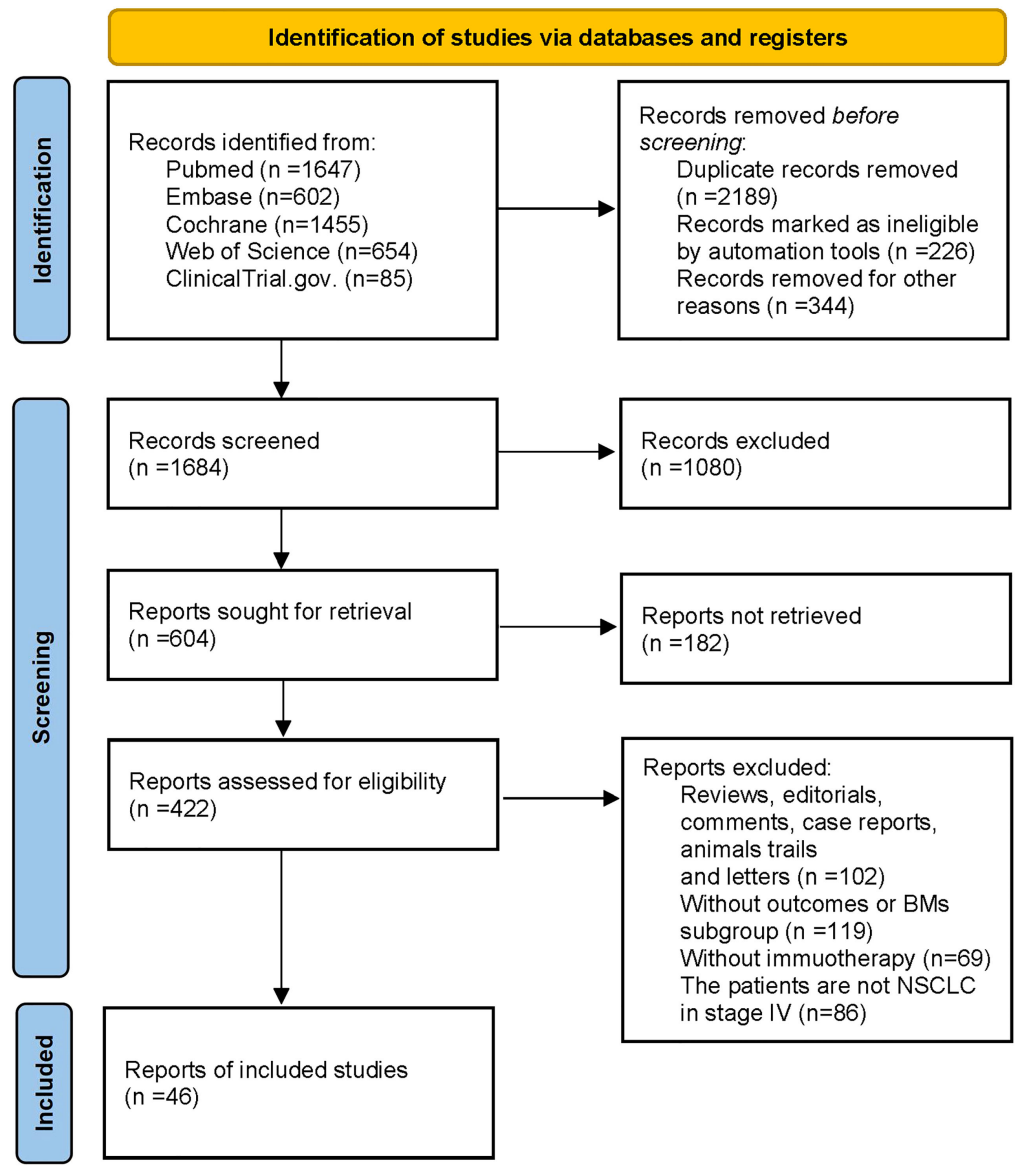
Flow chart of study selection in the meta-analysis.

Our literature identified 4443 studies initially, of which 2189 studies were regarded as duplicate records, 226 studies were marked as ineligible by automation tools, and 334 studies were removed for other reasons, such as lack of abstract. After screening the remaining 1684 abstracts, 1080 abstracts were excluded due to irrelevance. Following a retrieving process of relevant 604 records, we could not find 182 of them in publication. Among the remaining 422 articles, 102 were excluded for the article types (reviews, case reports, et al.), 119 did not include outcomes of BM subgroup, 69 were non-immunotherapy, and 86 articles contained results of other cancers such as melanoma. Subsequently, 46 studies were incorporated into the final analysis (27–29, 37–80). We illustrated the detailed process of the literature searches in a flow chart (**Figure 1**).

### General Characteristics of Included Studies

These included trials were heterogeneous, of which 22 were prospective, and 24 were retrospective. The immunotherapy group comprised patients treated with ICI monotherapy, ICI combined RT and ICI combined CT. Patients who were treated without ICI were incorporated into the immunotherapy-naive group. ICI monotherapy was defined as non-irradiation or non-chemotherapy 4 weeks prior to the ICI therapy, and the remaining immunotherapy groups were ICI combined RT or ICI combined CT group. To assess the efficacy of the sequencing of immunotherapy and radiotherapy on BM, ICI combined RT was divided into concurrent, RT before ICI, and RT after ICI.

Among the immunotherapy group, 23 arms were ICI monotherapy, nine were ICI combined CT, and 22 were ICI combined RT (**Figure S1A**), which contained 14 cohorts with RT before ICI, 12 concurrent, and four with RT after ICI (**Figure S1C**). Furthermore, seven groups received ICI combined SRS, one cohort received ICI combined WBRT, and the rest called mixed regimens did not differentiate between the two alternatives. But ICI combined RT groups were all analyzed in retrospective studies. Moreover, four dual ICI combined therapy were enrolled. A total of 33 trials were treated with PD-1 inhibitors. PD-1 combined CLTA-4 inhibitors were used in two studies. Four of the studies used PD-L1 inhibitors, and one involved PD-L1 and CLTA-4 inhibitors. Mixed regimens were classified as those in which the type of ICI was unknown, containing six trials.

We summarized participants’ sex, age, smoking history, EGFR/KRAS/ALK mutation, the expression of PD-L1, number and diameter of BM lesions, and pathological type in both **Table 1** and **Figure S1B**, and the efficacy in **Table S3**.

**Table 1:** Baseline of patients characteristics.

Author/year	Type of Study/Median follow-up time(mo)	Systemic therapy	Control	N of total pts/BM pts	% Sex (M)	Median age	% Smoking history	%EGFR/KRAS/ALK	%PD-L1 >1%	%Ad/Sq	XRT	N of mets (Total/median)	median max diameter of mets (cm)
Dudnik 2016 (38)	Retrospective/-	Nivo ± RT	–	5/5	40	70.2	80	0/40/-	–	60/20	unknown	–	–
Watanabe 2017 (39)	Retrospective/-	Nivo ± RT	–	48/19	–	–	–	–	–	–	unknown	–	–
Geier 2018 (40)	Retrospective/-	Nivo ± RT	–	77/77	72.7	57	90.9	-/29.8/-	–	72.7/-	unknown	–	–
Kobayashi 2018 (41)	Retrospective/-	Nivo ± RT	–	142/27	75	67	79.6	11.3/-/2.1	–	58.5/28.9	unknown	–	–
Hendriks 2019 (42)	Retrospective/15.8	Anti-PD-1± RT	–	1025/255	62	61.5	93.4	5.2/35.3/1	61.5	78/14.9	unknown	952/3.7	–
Zhang 2019 (43)	Retrospective/8.0	Nivo ± RT	–	73/32	78	57.7	–	9/3/-	–	75/22	unknown	66/2	–
Bjornhart 2019 (44)	Retrospective/15.7	Nivo/Pembro ± RT	–	118/21	47	66	94	3/-	82	69/23	unknown	–	–
Goldberg 2020 (45)	Prospective phase II/8.3	Pembro ± RT	Pembro	42/42	33	60	93	14/33/2	88	86/10/4	unknown	106/2.5	0.5-2
Checkmate0122020 (46)	Prospective phase I/-	Nivo	–	12/12	33	62.6	95	11/-/-	68	82/18	untreated	–	–
Ashinuma 2017 (47)	Retrospective/-	Nivo/Pembro	–	18/18	56	56	–	–	–	77/16	untreated	–	–
Henon 2017 (48)	Retrospective/17	ICI ± RT	–	259/48	–	63.1	87	5/26/1	28	64/-	unknown	–	–
Molinier 2017	Prospective phase III/22.1	Nivo	–	600/130	68	64	87	–	–	-/38	unknown	–	–
Dumenil 2018 (49)	Retrospective/-	Nivo ± RT	–	67/10	69	68.5	87	0/28/0	–	70/25	unknown	34/3.4	–
Gauvain 2018 (56)	Retrospective/5.8	Nivo ± RT	–	43/43	77	59.5	91	7/26	12	81/19	unknown	–	–
Wakuda1 2021	Retrospective/-	Pembro + RT	–	10/10	90	74.5	90	–	100	80/10	untreated	3.5 (1-10)	0.6 (0.2-1.64)
Wakuda2 2021 (51)	Retrospective/-	Pembro	–	13/13	62	69	92	–	100	62/23	treated	2 (1-10)	1.75 (0.6-6.6)
Lucio 2019 (52)	Prospective phase III/8.1	Nivo ± RT	–	1588/409	65	63	74	–	–	–	unknown	–	–
Cortinovis 2019 (53)	Prospective phase III/7.1	Nivo ± RT	–	371/37	65	64	79	–	–	–	unknown	–	–
Achim 2016 (28)	Prospective phase III/21	Atezolizumab	Chemo	850/85	61	63	80	10/6/<1	57	74/26	treated	–	–
Ahmet 2021 (57)	Prospective phase III/10.8	Cemiplimab	Chemo	563/34	88	63	100	–	100	57/43	treated	–	–
Lu 2021 (55)	Prospective phase III/25.9	Nivo	Chemo	504/45	78	60	70	–	55	61/39	untreated	–	–
Goldman 2020 (59)	Retrospective/8.4	Nivo	Chemo	46/46	–	–	–	–	–	–	unknown	–	–
Borghaei 2015 (60)	Prospective phase III/13.2	Nivo	Chemo	582/34	55	62	78.7	14/11/4	53	93/0.8	treated	–	–
Martin 2019 (61)	Prospective phase III/25.2	Pembro	Chemo	305/18	59.7	64.5	96.8	–	–	82/18	treated	–	–
Mansfield 2019 (62)	Retrospective/18.4	pembro	Chemo	3170/199	–	–	–	–	100	–	unknown	–	–
Matthew 2019 (67)	Prospective phase III/24	Nivo + Ipilimumab	Chemo	1166/81	66.7	64	85.4	–	68	70.5/27.9	treated	–	–
Muhammad 2018 (63)	Retrospective/-	Pembro + Chemo	Chemo	54/6	44	65	93	–	53.6	100/0	unknown	–	–
Powell 2019 (54)	Retrospective/10.9	Pembro + Chemo	Chemo	616/73	62	65	88.3	–	63.4	96.1/2.4	unknown	–	–
Caicun 2020 (79)	Prospective phase III/11.9	Camrelizumab+Chemo	Chemo	412/11	71	59	62.4	none	67	99/0	treated	–	–
Yunpeng 2020 (65)	Prospective phase III/8.9	Sintilimab +Chemo	Placebo+Chemo	397/36	76.7	61	64.3	none	68	95.1/0	untreated	–	–
Shepard 2019 (13)	Retrospective/-	SRS + ICI	SRS	17/17	61	64	–	–	71.4	–	treated	45/2.6	1.0 (0.2-1.8)
Singh 2019 (66)	Retrospective/12	ICI + SRS	Chemo+SRS	39/39	41	62	–	–	–	68/10.6	treated	291/7.5	–
Patruni 2019 (68)	Retrospective/11.4	ICI+RT	RT	545/545	–	–	–	–	–	–	treated	–	–
Enright 2020 (69)	Retrospective/9.87	ICI+RT	RT	33/33	61	62	–	–	–	74/19	treated	64/2 (1-5)	0.65 (0.04-6.5)
Imber 2017 (70)	Retrospective/3.9	ICI+RT	–	45/45	–	–	–	–	–	84/-	treated	91/2 (1-4)	0.8 (0.1-4)
Srivastava 2018 (71)	Retrospective/14.3	ICI+RT	–	42/42	–	–	–	–	–	–	treated	–	–
Ahmed 2017 (72)	Retrospective/8.7	ICI+SRS	–	17/17	58.8	60	–	11.8/17.6/-	–	–	treated	49/3 (1-4)	0.57 (0.2-2.3)
Gandhi 2018 (73)	Prospective phase III/10.5	pembro + Chemo	Chemo	410/73	62.0	65	88.3		63.4	96.1/2.4	untreated	–	–
Schapira 2018 (37)	Retrospective/14.3	SRS + ICI	–	37/37	35.1	63	–	–	–	–	treated	85/2 (1-3)	0.6 (0.2-2.6)
Wu 2019 (58)	Prospective phase III/10.4	Nivo	Chemo	338/45	77.8	60	69.8	–	49.7	60.1/39.3	untreated	–	–
Fehrenbacher 2018 (74)	Prospective phase III/27	Atezolizumab	Chemo	613/118	61.8	63	81.7	9.8/6.9/0.7	56.6	73.7/26.3	treated	–	–
Ernest 2021 (75)	Prospective phase II/17.3	Atezolizumab+Chemo		40/40	72.5	62.6	75	–	–	97.5/2.5	untreated	–	–
David 2021 (76)	Prospective phase III/12.7	Nivo + Ipilimumab+Chemo	Chemo	719/64	70	65	87	–	60	69/31	treated	–	–
Wang 2021 (77)	Prospective phase II/12	Sintilimab+Chemo		40/10	77.5	55	55	10/15/-	12.5	87.5/12.5	unknown	–	–
Miranda 2021 (80)	Prospective phase III/16.3	Cemiplimab+Chemo	Chemo	312/24	85.9	63	30.8	–	69.5	57.4/42.6	unknown	–	–
Caicun 2021 (64)	Prospective phase III/8.6	Sugemalimab+Chemo	Chemo	320/50	79.4	62	72.5	–	61.3	59.7/40.3	unknown	–	–
Natasha 2021 (78)	Prospective phase/16.6	Durvalumab + Tremelimumab +Chemo	Durvalumab + Tremelimumab	301/49	90	64	54	–	47	82/18	unknown	–	–

BM, brain metastases; F, female; mo, months; pts, patients; N, number; ICI, immune checkpoint inhibitors; Nivo, nivolumab; Pembro, pembrolizumab; Chemo, chemotherapy; q2w, every two weeks; q3w, every 3 weeks; Ad, adenocarcinoma; Sq, squamous-cell carcinoma; SRS, stereotactic radiosurgery; con, concurrent; seq, sequential.

### Pool-Analysis of the Efficacy of Different Immunotherapy Systematic Regimens

**Figure 2:**
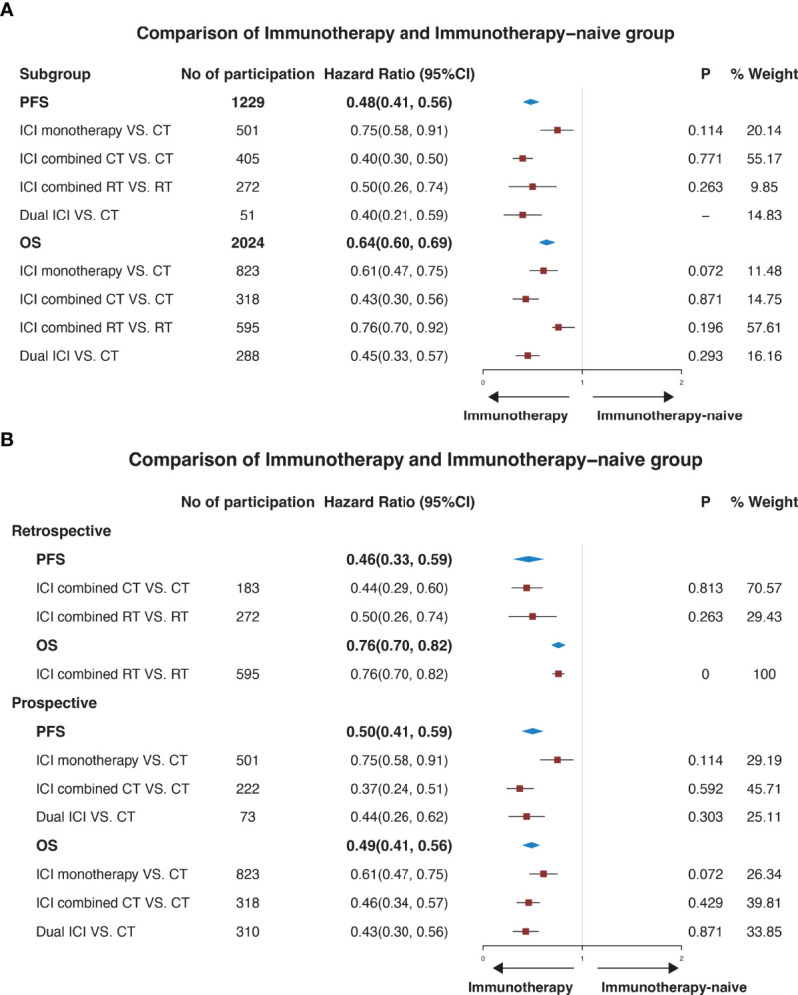
Forest plots illustrating pooled results of efficacy for the comparison of patients with or without immune checkpoint inhibitors. The immunotherapy group was defined as patients who received ICI. The immunotherapy-naive group was defined as patients who didn’t receive ICI. **(A)** illustrates a pooled result in all studies, and **(B)** illustrates a pooled result of subgroup analysis according to the type of study. No, number; CI, confidence interval; P, p-value of heterogeneity. PFS, progression-free survival. OS, overall survival; CT, chemotherapy; RT, radiotherapy.

Strong evidence in **Figure 2A** showed that when compared with the ICI naive group, the immunotherapy group was associated with significantly longer PFS (0.48, 95% CI: 0.41-0.56) and OS (0.64, 95%CI: 0.60-0.69). 501 patients with BM were included to evaluate PFS of ICI monotherapy against CT, and 823 patients were enrolled to compare OS. The HRs of PFS and OS were 0.75 (0.58, 0.91) and 0.61 (0.47-0.75). The HR of ICI combined CT versus CT was 0.40 (95%CI: 0.30-0.50), and 0.43 (95%CI: 0.30-0.56). The HRs were 0.50 (0.26-0.74) and 0.76 (0.70-0.92) respectively in ICI combined RT versus RT. A significant difference was also observed between immunotherapy and immunotherapy-naive groups in the retrospective and prospective studies respectively. The HR of PFS was 0.46 and OS was 0.76 in retrospective studies, which was 0.50 and 0.49 in prospective studies (**Figure 2B**).

**Figure 3:**
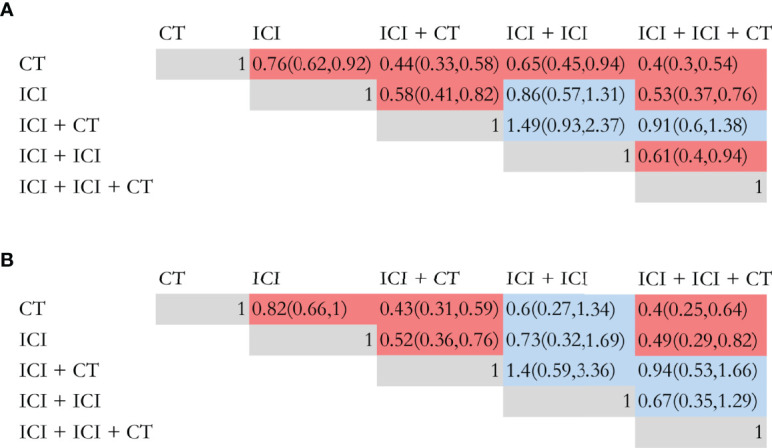
Efficacy of different immune checkpoint inhibitor regimens profiles based on overall survival **(A)** and progression-free survival **(B)**. Each cell of the efficacy profiles contains the pooled hazard ratio and 95% confidence intervals; significant results are in red, otherwise, in blue. The pooled hazard ratio and 95% confidence intervals indicate the results of the column name compared with the row name. ICI, immune checkpoint inhibitor; CT, chemotherapy.

A network meta-analysis was conducted in prospective studies to compare the efficacy of various ICI systemic regimens for the treatment of NSCLC patients with BM (**Figure S2**). In terms of OS (**Figure 3A**), ICI monotherapy, ICI combined CT, dual ICI, and dual ICI combined CT exhibited a relatively better efficacy compared to CT (HR = 0.76, 95%CI = 0.62-0.92; HR = 0.44, 95%CI = 0.33-0.58; HR = 0.65, 95%CI = 0.45-0.94; HR =0.4, 95%CI =0.3-0.54, respectively). Dual ICI combined CT and ICI combined CT achieved the highest OS. When compared to ICI monotherapy, the HR of ICI combined CT was 0.58, and the HR of dual ICI combined CT was 0.53. Furthermore, the HR between dual ICI combined CT and dual ICI was 0.61(0.4, 0.94).

As shown in **Figure 3B**, ICI monotherapy, ICI combined CT, and dual ICI combined CT were all more effective than CT (HR = 0.83, 95%CI = 0.66-1; HR = 0.43, 95%CI = 0.31-0.59; HR = 0.4, 95%CI = 0.25-0.64). There was no difference between dual ICI and CT, and only Natasha (73) investigated the efficacy of dual ICI (durvalumab plus tremelimumab), which might cause bias in the comparison. The optimal for enhancing PFS were also dual ICI combined CT and ICI combined CT, of which the HR was 0.4 and 0.43, respectively.

### Pool-Analysis of the Efficacy of ICI Monotherapy Versus ICI Combined Radiotherapy

**Figure 4:**
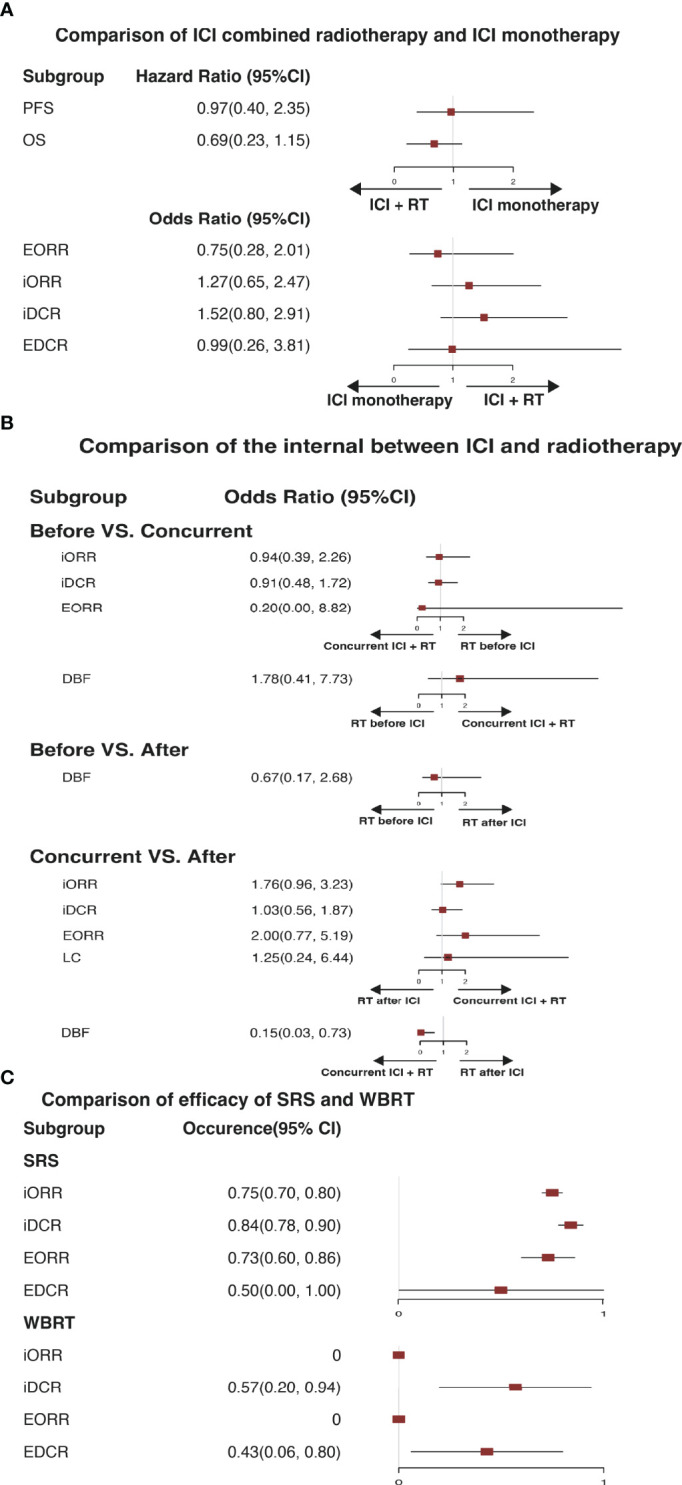
**(A)** Illustrates a pooled therapeutic result in comparison of immune checkpoint inhibitor monotherapy and immune checkpoint inhibitor combined radiotherapy. **(B)** Illustrates a pooled therapeutic result of comparison among radiotherapy before immune checkpoint inhibitor, concurrent immune checkpoint inhibitor combined radiotherapy, and radiotherapy after immune checkpoint inhibitor. **(C)** Illustrates a pooled result in comparison of stereotactic radiation surgery and whole-brain radiation therapy. ICI, immune checkpoint inhibitor; RT, radiotherapy; SRS, stereotactic radiation surgery; WBRT, whole-brain radiation therapy; PFS, progression-free survival. OS, overall survival; iORR, intracerebral objective response rate; iDCR, intracerebral disease control rate; EORR, extracranial overall response rate; EDCR, extracranial disease control rate; DBF, distant brain failure; LC, local control.

The standard treatment for NSCLC patients with BM was cranial radiation previously, so the comparison was performed to analyze the effectiveness of ICI combined RT and ICI monotherapy (**Figure 4**). Surprisingly, there was no statistical difference in PFS (HR=0.97, 95%CI: 0.40-2.35), OS (HR=0.69, 95%CI: 0.23-1.15), EORR (OR=0.75, 95%CI: 0.28-2.01), iORR (OR=1.27, 95%CI: 0.65-2.47), iDCR (OR=1.52, 95%CI: 0.80-2.91), and EDCR (OR=0.99, 95%CI: 0.26-3.81) for patients with ICI combined intracranial radiation or ICI monotherapy (**Figure 4A**).

Therefore, we hypothesized whether the sequencing of immunotherapy and RT affected the efficacy of ICI combined RT. Subsequently, a subgroup meta-analysis of three radiotherapy regimens, RT before ICI (BEFORE), concurrent RT and ICI (CONCURRENT), and RT after ICI (AFTER), was conducted (**Figure 4B**). In the comparison of Before and Concurrent groups, no obvious differences were observed in iORR, iDCR, EORR, and DBF (OR = 0.94, 95%CI: 0.39-2.26; OR = 0.91, 95%CI: 0.48-1.72; OR = 0.20, 95%CI: 0.00-8.82; OR = 1.78, 95%CI: 0.41-7.73, separately). The DBF of BEFORE versus AFTER group was 0.67, ranging from 0.17 to 2.68, indicating no difference. A discernible difference between CONCURRENT and AFTER was shown in DBF (OR = 0.15, 95%CI: 0.03-0.73), which demonstrated that concurrent treatment was associated with favorable locoregional disease control.

For NSCLC patients with BMs, WBRT and SRS are the preferred intracranial radiation treatments. In our study, seven trials assessed the short-term efficacy of ICI combined SRS or WBRT (**Figure 4C**). Referring to ICI combined SRS, the iORR, iDCR, EORR, and EDCR were 75%, 84%, 73%, and 50%, separately. In terms of ICI combined WBRT, these were 0%, 57%, 0%, and 43%, separately.

### Pool-Analysis of the Efficacy in Different ICI Type

**Figure 5:**
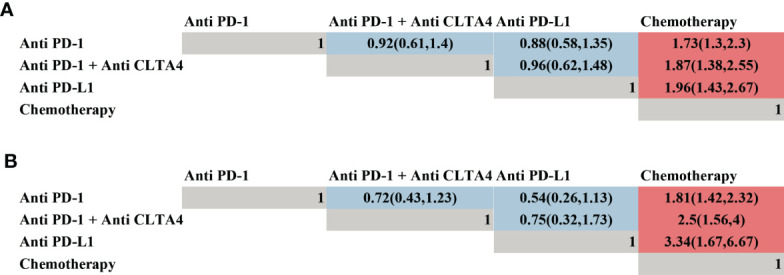
Efficacy of different types of immune checkpoint inhibitor profiles based on overall survival **(A)** and progression-free survival **(B)**. Each cell of the efficacy profiles contains the pooled hazard ratio and 95% confidence intervals; significant results are in red, otherwise, in blue. The pooled hazard ratio and 95% confidence intervals indicate the results of the column name compared with the row name.

Moreover, a network meta-analysis was performed to compare the efficacy of PD-1, PD-L1, and PD-1 combined CLTA4 inhibitors (**Figure 5**). As shown in **Figure 5A**, PD-1, PD-L1, and PD-1 combined CLTA4 inhibitors all demonstrated a better OS (**Figure 5A**) and PFS (**Figure 5B**) in comparison with CT. However, no significant difference was observed among the three types of ICI for treating BM, which might indicate that they were all potential choices.

### Sensitivity Analysis and Risk of Bias

Sensitivity analysis was performed by excluding one individual study each time to assess the influence of each individual study on the pooled HRs for OS or PFS. The omission of any single study did not appreciably change the pooled HR, and the estimates in each case were well within the confidence limits of the overall estimate (**Figure S4**). The NOS results are listed in **Table S2**. **Figure S5** provides the Cochrane risk of bias. Thus, our meta-analysis revealed a positive correlation between immunotherapy and the prognosis of NSCLC patients with BM.

## Discussion

NSCLC patients complicated with BMs, especially those whose driver genes mutations are negative or TKI resistant, remain a challenge to treat. However, the roles of ICI regimens for those patients are not completely established. We, therefore, summarized the ICI regimens in NSCLC patients with BMs and performed a meta-analysis to provide a theoretical basis for future treatment strategies. In total, we included 46 articles with 3160 NSCLC patients with BMs. Twenty-two articles were prospective studies, and 24 articles were retrospective studies. Three findings were yielded in our study.

Firstly, immunotherapy may be preferable over non-immunotherapy for NSCLC patients with BMs, with longer PFS (HR = 0.48) and OS (HR = 0.64). The advantage may be caused by the synergy between immunotherapy and chemotherapy/radiotherapy (81–86). For example, immunotherapy enhanced the radiotherapy-induced abscopal effect and reversed the immunosuppressive effect of radiation, by blocking the immune checkpoint between antigen-presenting cells and lymphocytes in regional lymph nodes and other organs. Meanwhile, radiation destroyed the endothelial junctions of the BBB, promoted tumor antigen release, and up-regulated T-cell mediated immune response and PD-L1 expression, which in turn prompted the efficacy of immunotherapy. Simultaneously, chemotherapy promoted tumor immunity by inducing immunogenic cell death as part of its intended therapeutic effect and disrupting strategies that tumors use to evade immune recognition. Moreover, CTLA-4 can impair the critical signal transmission of T-cell activation by competitively inhibiting the CD28 receptor’s binding to B-7 ligands, and PD-1 blocks T-cell activation directly. ICI could inhibit the two pathways so that active peripheral immune cells could penetrate BBB. Our findings were consistent with the conclusions of real-world clinical studies, which showed that immunotherapy had a therapeutic benefit in NSCLC patients with BM.

Secondly, no significant difference in PFS (HR = 0.97, 95%CI: 0.40-2.35), OS (HR = 0.69, 95%CI: 0.23-1.15), EORR (OR = 0.75, 95%CI: 0.28-2.01), iORR (OR = 1.27, 95%CI: 0.65-2.47), iDCR (OR = 1.52, 95%CI: 0.80-2.91), and EDCR (EORR (OR = 0.99, 95%CI: 0.26-3.81) was observed between ICI combined RT and ICI monotherapy, which is consistent with Alencar’s study (30). The difference in PD-L1 expression or lymphocyte tumor infiltration might be the biological mechanism of variable response rates on ICIs for BMs (87, 88). However, there were insufficient data to assess survival outcomes and stratify response based on other important factors like the time of BM diagnosis (newly diagnosed or recurrent), the number, size, and location of metastases, the presence of extracranial disease, the presence of actionable driver gene mutations, and PD-L1 expression.

Interestingly, the sequencing of immunotherapy and RT in the treatment of BM from NSCLC might influence the efficacy of ICI combined RT. Concurrent ICI combined RT might have a lower recurrence rate than sequential ICI combined RT, especially RT after ICI, with a DBF of 0.15. However, no significant improvement in efficacy was found in concurrent ICI combined RT, like iORR, iDCR, EORR, or EDCR, which is inconsistent with previous studies. The interval of ICI and cranial RT might be the core of inconsistency. The largest published retrospective study of patients receiving concurrent immuno-combined RT, the interval of which was shorter than 2 weeks, had a significantly longer OS, reduced incidence of new BM lesions, and an acceptable safety profile (89). The latest prospective study by Wang (unpublished, NCT02978404), a phase II study, in which the interval between nivolumab and SRS was also 2 weeks, achieved a median intracranial PFS of 6.5 months, and an OS of 21.4 months. Coincidentally, in the Emory trial of Khan (unpublished, NCT02858869), the interval of which between pembrolizumab and SRS was 2 to 3 days, also reached a median intracranial PFS of 7.5 months, and an OS of 32.8 months. In addition, a prospective study (unpublished, NCT02696993) that adopted concurrent SRS with dual immunotherapy (Nivolumab and Ipilimumab) with a 7-day interval, achieved a median intracranial PFS of 9.7 months, and a 4-month intracranial PFS rate of 75%. In combination with our analysis and the positive results of ongoing prospective trials, it provides strong support for the efficacy of concurrent ICI combined with RT in 4 weeks (90, 91), which is the drug wash-out period and the destruction of the BBB endothelial cell generated by radiotherapy, for NSCLC patients with BM.

Moreover, when evaluating the efficacy and tolerability of ICI combined RT for BM, it’s important to consider the variety of radiation treatment modalities and dose fractionation prescriptions used. Initial analysis suggested SRS achieved an obvious increase in iORR and EORR, compared to WBRT, which were 75% versus 0 and 73% versus 0, respectively. It was possibly connected to dosage distribution. WBRT delivers the same modest palliative radiation dosage to healthy brain tissue (non-ablative). While SRS delivers a strong ablative dosage solely to metastatic tissue (92). In our included research, the average number of BM was two, the diameter of which ranged from 0.5 to 2 cm, which was defined as small oligometastases. However, WBRT might be suitable for diffuse BM, and it had more acute toxicities than SRS, increases fatigue, lowers the quality of life, and impairs cognitive function (93). Our results correspond to multiple clinical studies, which indicated SRS might eventually replace WBRT for patients with localized (1-3) minor lesions (less than 4 cm in size).

Thirdly, dual ICI combined CT and ICI combined CT provided a better PFS and OS. Compared to dual ICI, the HRs of dual ICI combined CT were 0.61 and 0.67. The HRs of ICI combined CT versus ICI were 0.58 and 0.52, respectively. Surprisingly, there was no difference between dual ICI and ICI combined CT. The mechanisms and efficacy of ICI combined CT varied by different CT agents. Such as platinum-based CT, on the one hand, its favorable immunomodulation effects may boost tumor cells’ susceptibility to PD-1/PD-L1 inhibitors. On the other hand, down-regulation of intracellular PD-L1 expression by PD-1/PD-L1 inhibitors could make patients sensitive to platinum-based CT. However, the mechanisms and efficacy of ICI combined with CT varied by different chemotherapy agents. A dual ICI regimen was defined as a combined blockade of PD-1/L1 and CTLA-4, which offers a number of advantages over a single PD-1 inhibitor without the limitation of BBB. Firstly, PD-1/L1 inhibition is linked to CTLA-4 overexpression, thus anti-CTLA-4 inhibitors might prevent further immune escape directly. Secondly, myeloid-derived suppressor cells can severely limit T cell function inside the tumor microenvironment, but dual ICI can raise the fraction of CD8+ effector T cells relative to MDSCs synergistically. Thirdly, dual ICI could raise inflammatory cytokine production, such as TNF-α and IFN-γ, while lowering T cell anergy. Finally, dual ICI could lead to the growth of memory T cells, which facilitates longer-term anti-tumor immunity (94). But the brain was an immune-specialized environment, in which immune responses against tumors were restricted. Taken together, these confounding factors might weaken the difference of efficacy of ICI combined CT and dual ICI. After combining the results of dual ICI combined CT and ICI combined CT in our study, the ranking of efficacy and recommendation based on the five treatment groups were as follows: dual ICI combined CT, ICI combined CT, dual ICI, ICI monotherapy, and CT.

Another aspect that determines the effectiveness of ICIs is the choice of PD-1, PD-L1, or CTLA-4 inhibitors. Even though Duan et al. (95) found PD-1 inhibitors had better OS and PFS than PD-L1 inhibitors in various cancers, it’s uncertain if the three forms of ICIs, PD-1, PD-L1, and CLTA-4 inhibitors in NSCLC patients with BMs have different intracranial activity. Our analysis found no statistical difference in PD-1, PD-1 combined CLTA-4, and PD-L1 inhibitors for the first time. Still, further exploration was warranted to elucidate the specific mechanism.

Our strengths involved a comprehensive, systematic review of studies by a multidisciplinary team including specialists in NSCLC with BM and epidemiological methods. A broad search strategy was employed to catch all relevant studies. Therefore, our analysis was more comprehensive than other literature with similar topics. Furthermore, there was no proof of publication bias. Importantly, our study not only was the first meta-analysis concerning different ICI regimens, but also the first to compare the intracranial efficacy of WBRT and SRS, and three different ICI types for NSCLC patients with BM.

Our limitations deserve comments. In the analysis of ICI monotherapy versus ICI combined RT, most studies were retrospective, which makes pairwise analysis unavailable, and more prone to selection bias. Despite that, the results that ICI monotherapy might be effective as a single treatment for patients with BM were consistent with, and replenish, Alencar’s therapeutic perspective.

In conclusion, ICI should be considered for selected individuals lacking actionable driver gene mutations. Furthermore, concurrent ICI combined RT in 4 weeks demonstrated improved DBF, and SRS was superior to WBRT for localized and tiny BMs. Our findings revealed that dual ICI combined CT and ICI combined CT had better OS and PFS, giving possible efficacy speculations for clinical decisions. More prospective clinical studies evaluating the benefit of PD-1, PD-L1, and CTLA-4 inhibitors are required in the future, to elucidate why no significant difference in the efficacy of three distinct types of ICI was identified.

## Data Availability Statement

The original contributions presented in the study are included in the article/**Supplementary Material**. Further inquiries can be directed to the corresponding authors.

## Author Contributions

All authors were involved in the design of the study, organization of the study, manuscript writing, and approval of final version of the manuscript. XC and LN generated the search strategy, performed the search, independently performed the article selection procedure, data extraction, and summarized the data. ZZL performed the data analyses, and RZ checked the data analysis and validity. XC and LN wrote the manuscript, had contributed equally to the manuscript. RZ and ZZL had contributed equally to the manuscript. All authors contributed to the article and approved the submitted version.

## Funding

This study was supported by Beijing Xisike Clinical Oncology and Research Foundation (Y-HR2019-0185) and National Multidisciplinary Cooperative Diagnosis and Treatment Capacity Building Project for Major Diseases of China (z027002).

## Conflict of Interest

The authors declare that the research was conducted in the absence of any commercial or financial relationships that could be construed as a potential conflict of interest.

## Publisher’s Note

All claims expressed in this article are solely those of the authors and do not necessarily represent those of their affiliated organizations, or those of the publisher, the editors and the reviewers. Any product that may be evaluated in this article, or claim that may be made by its manufacturer, is not guaranteed or endorsed by the publisher.
