# Supplementary figures and images for: The Long-Term and Short-Term Efficacy of Immunotherapy in Non-Small Cell Lung Cancer Patients With Brain Metastases: A Systematic Review and Meta-Analysis
Source: Front Immunol. 2022 May 25;13:875488. doi: 10.3389/fimmu.2022.875488 (PMC9175180; doi:10.3389/fimmu.2022.875488)

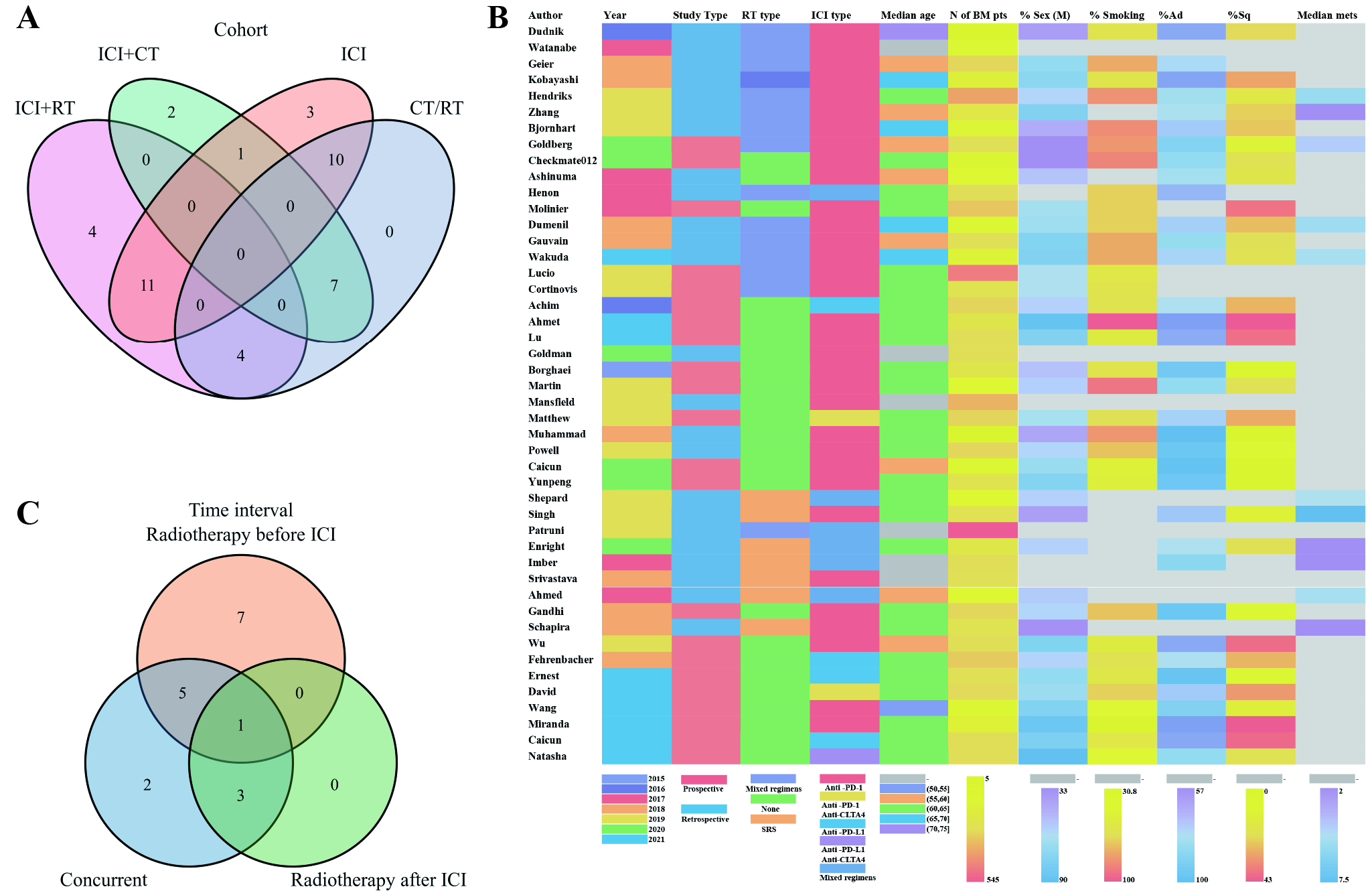

Supplement: Supplementary file 1 [file Image_1.tif]

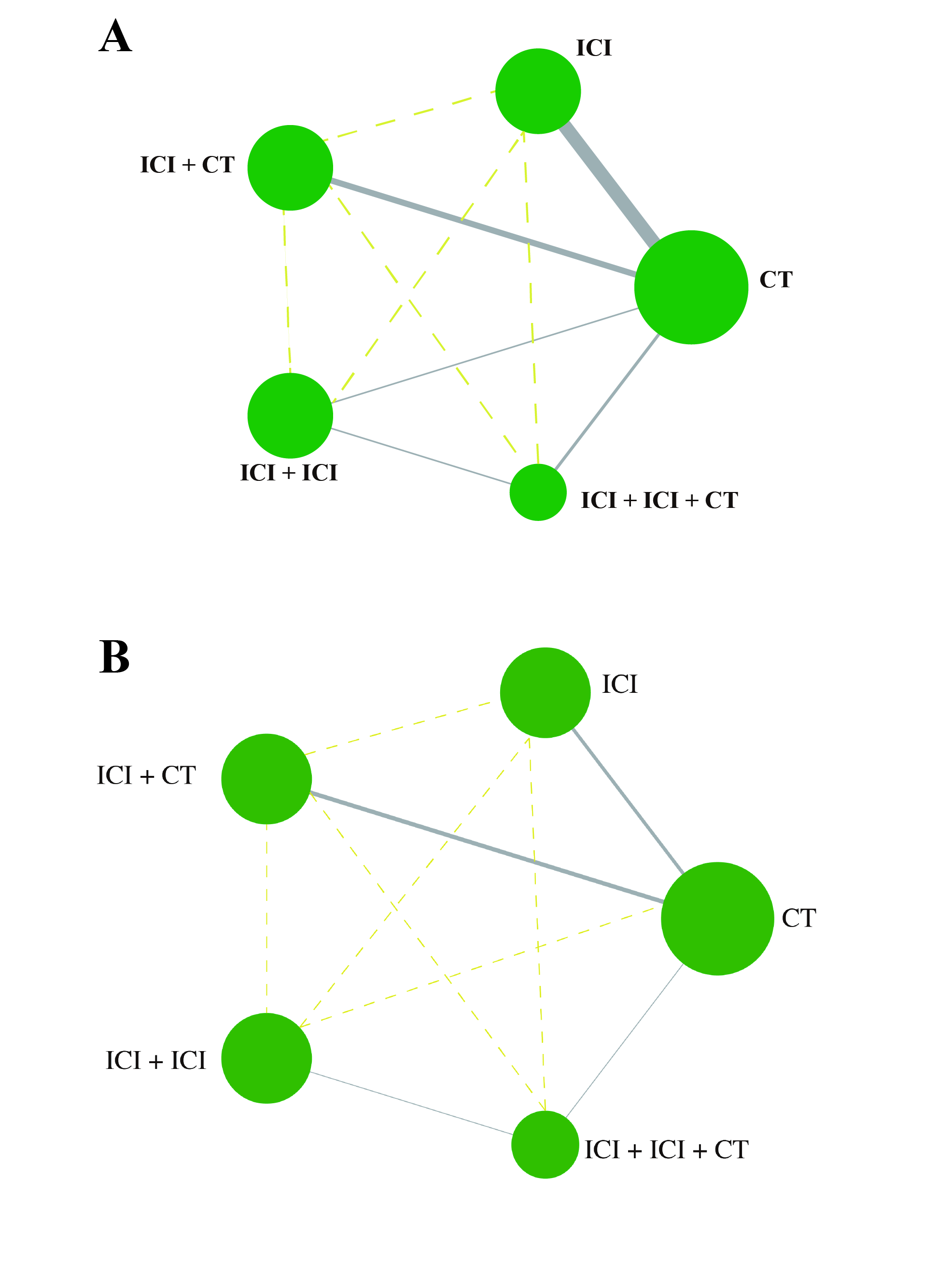

Supplement: Supplementary file 2 [file Image_2.tif]

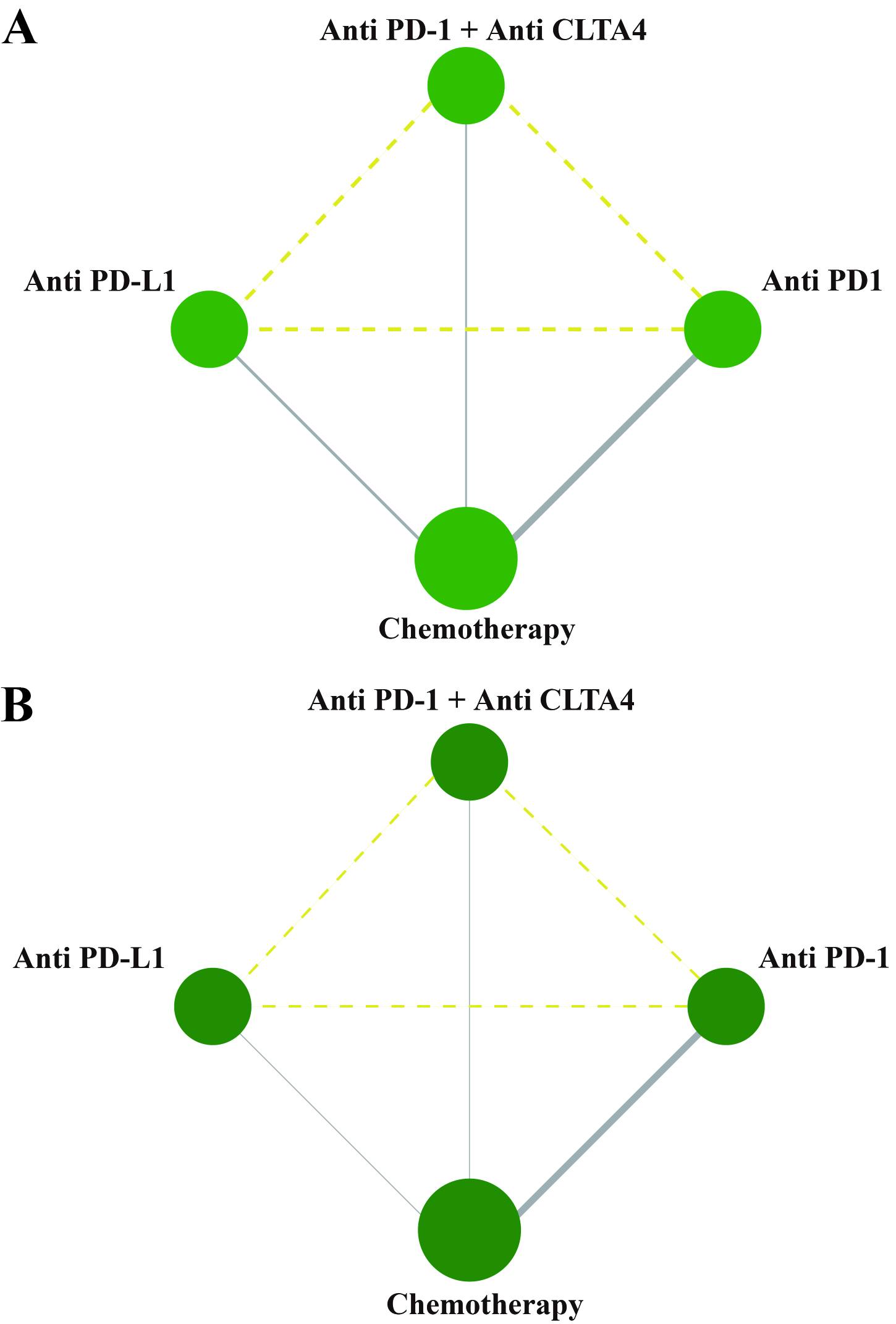

Supplement: Supplementary file 3 [file Image_3.tif]

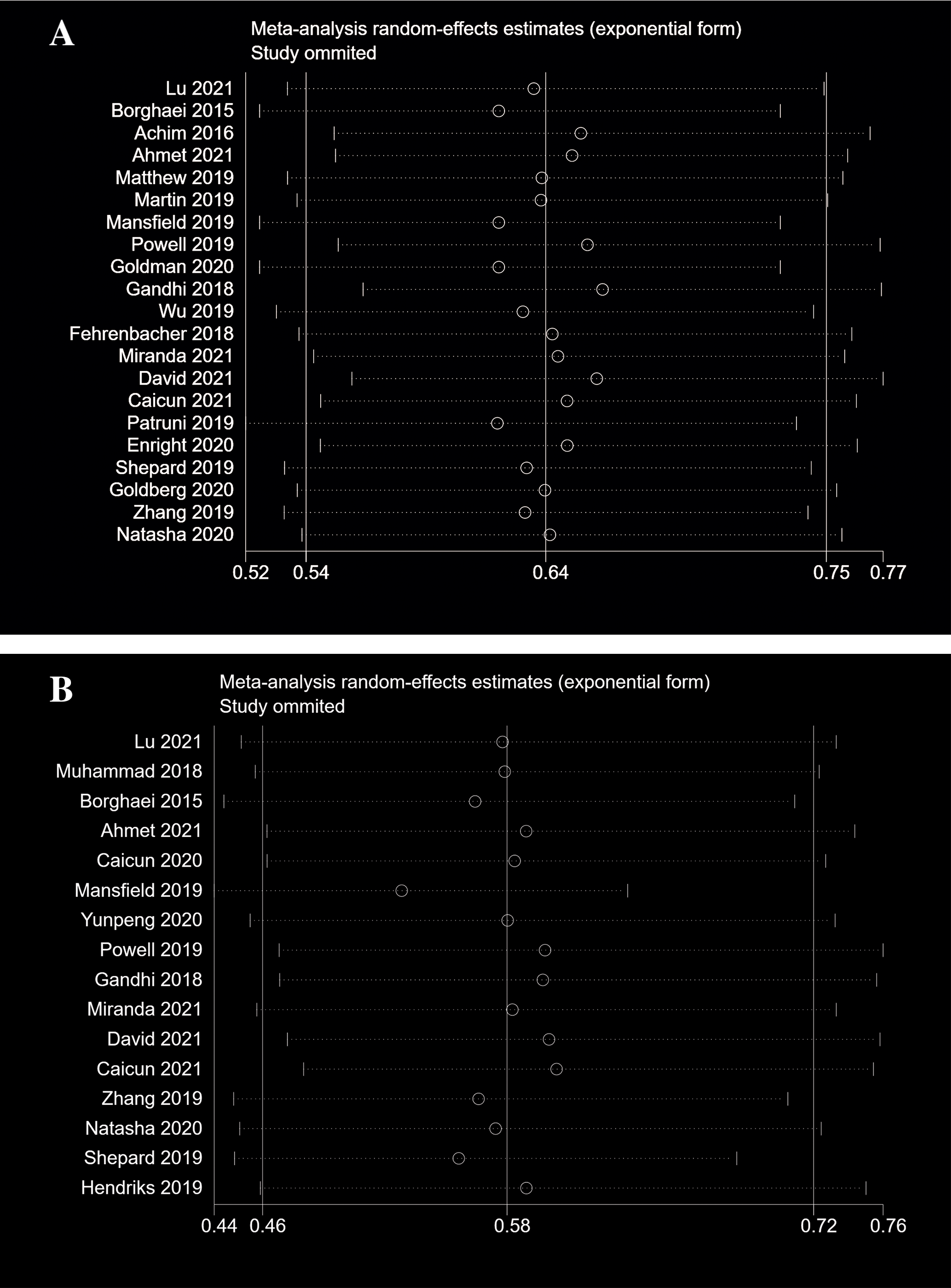

Supplement: Supplementary file 4 [file Image_4.tif]

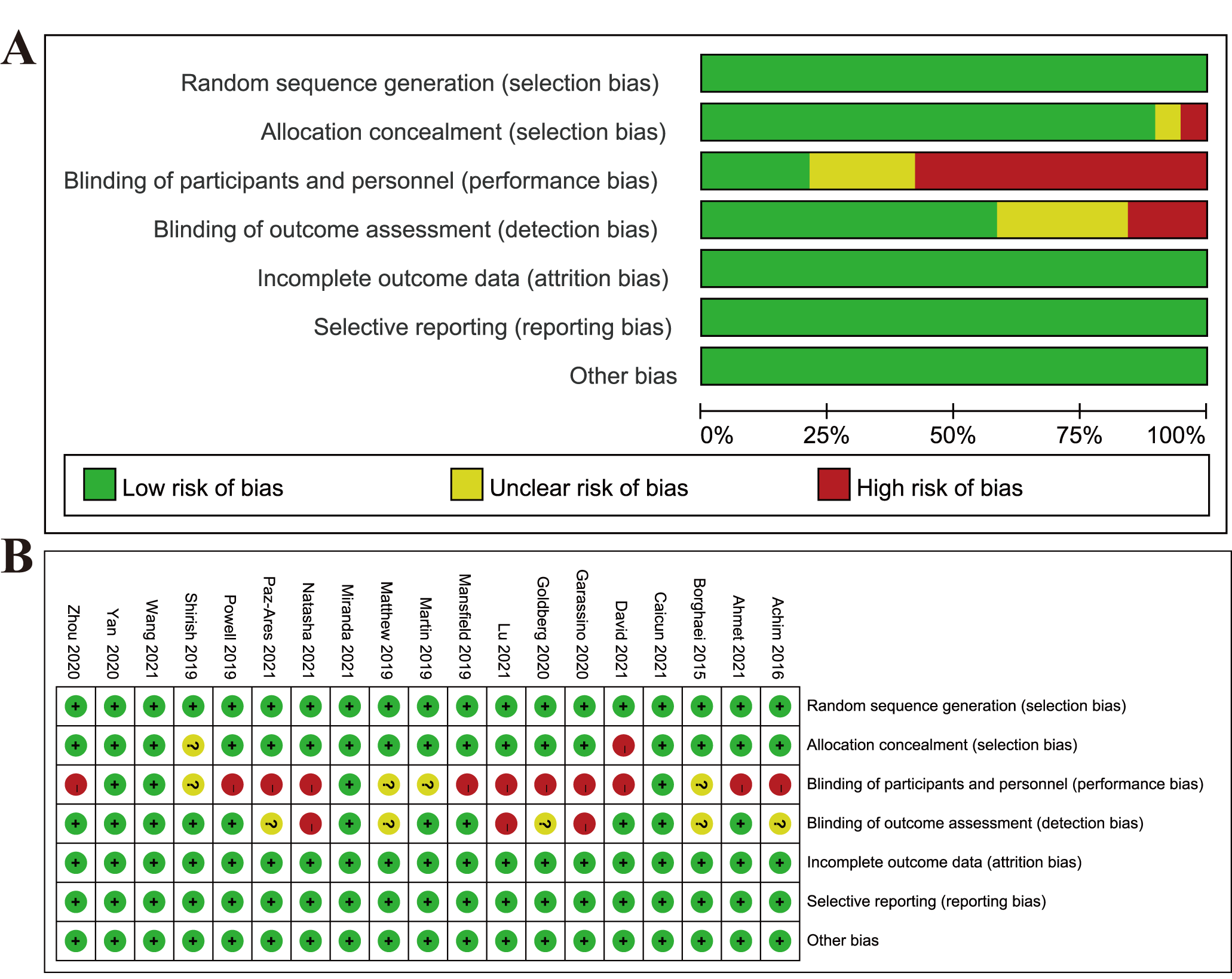

Supplement: Supplementary file 5 [file Image_5.tif]
