# Supplementary material for: The Long-Term and Short-Term Efficacy of Immunotherapy in Non-Small Cell Lung Cancer Patients With Brain Metastases: A Systematic Review and Meta-Analysis
Source: Front Immunol. 2022 May 25;13:875488. doi: 10.3389/fimmu.2022.875488 (PMC9175180; doi:10.3389/fimmu.2022.875488)
Supplement: Supplementary file 7 [file Table_2.docx]

**Table S2** Methodological quality assessment (risk of bias) of included non-RCT studies by Newcastle-Ottawa Scale

| **Study** | **Selection** | | | | **Comparability** | **Outcome** | | | **Total score** |
| --- | --- | --- | --- | --- | --- | --- | --- | --- | --- |
|  | Exposed Cohort | Nonexposed Cohort | Ascertainment of exposure | Outcome of interest |  | Assessment of outcome | Length of follow-up | Adequacy of follow-up |  |
| Dudnik 2016 | * | - | * | * | - | * | * | * | 6 |
| Watanabe 2017 | * | * | * | * | * | * | * | * | 8 |
| Ashinuma 2017 | * | * | * | * | * | * | * | * | 8 |
| Henon 2017 | * | * | * | * | * | * | - | * | 7 |
| Dumenil 2018 | - | * | * | * | * | * | * | * | 7 |
| Kobayashi 2018 | * | * | * | * | * | * | * | * | 8 |
| Geier 2018 | * | * | * | * | ** | * | - | * | 8 |
| Hendriks 2019 | * | * | * | * | ** | * | * | * | 9 |
| Zhang 2019 | * | * | * | * | ** | * | - | * | 8 |
| Bjørnhart 2019 | * | * | * | * | ** | * | * | * | 9 |
| Checkmate 012-arm M 2020 | * | - | * | * | * | * | * | * | 7 |
| Muhammad 2018 | * | * | * | * | * | * | * | * | 8 |
| Wakuda 2021 | * | * | * | * | ** | * | - | * | 8 |
| Shepard 2019 | * | * | * | * | ** | * | * | * | 9 |
| Gauvain 2018 | * | * | * | * | ** | * | - | * | 8 |
| Cortinovis 2019 | * | * | * | * | ** | * | * | * | 9 |
| Molinier 2017 | * | - | * | * | * | * | * | * | 7 |
| Signh 2019 | * | * | * | - | ** | * | * | * | 8 |
| Goldman 2016 | * | * | * | - | ** | * | * | * | 8 |
| [Lucio](https://pubmed.ncbi.nlm.nih.gov/?term=Crin%C3%B2+L&cauthor_id=30797489) 2019-1 | * | * | * | * | ** | * | * | * | 9 |
| Lucio 2019-2 | * | * | * | * | * | * | * | * | 8 |
| Patruni 2019 | * | * | * | * | * | * | * | * | 8 |
| Enright 2020 | * | * | * | * | ** | * | * | * | 9 |
| Imber 2017 | * | * | * | * | ** | * | * | * | 9 |
| Srivastava 2018 | * | * | * | * | ** | * | * | * | 9 |
| Ahmed 2017 | * | * | * | * | ** | * | * | * | 9 |
| Schapira 2018 | * | * | * | * | ** | * | * | * | 9 |
| Ernest 2021 | * | * | * | * | * | * | * | * | 8 |
