# Supplementary material for: The Long-Term and Short-Term Efficacy of Immunotherapy in Non-Small Cell Lung Cancer Patients With Brain Metastases: A Systematic Review and Meta-Analysis
Source: Front Immunol. 2022 May 25;13:875488. doi: 10.3389/fimmu.2022.875488 (PMC9175180; doi:10.3389/fimmu.2022.875488)
Supplement: Supplementary file 8 [file Table_3.docx]

Table S3 Efficacy of enrolled patients

| Author/Year | Arm | EORR, % | iORR, % | EDCR, % | iDCR, % | DBF, % | LC, % | PFS (HR) | OS (HR) | Response Assessment Method |
| --- | --- | --- | --- | --- | --- | --- | --- | --- | --- | --- |
| Dudnik 2016 | concurrent | 100 | 100 | - | 100 | - | - | - | - | mRECIST 1.1 |
|  | sequential | 0 | 0 | - | 50 | - | - | - | - |  |
|  | -XRT | 50 | 50 | - | 50 | - | - | - | - |  |
| Watanabe 2017 | +XRT | 13 | - | 20 | - | - | - | - | - | NS |
|  | -XRT | 0 | - | 25 | - | - | - | - | - |  |
| Geier 2018 | sequential | 20.8 | 6.7 | - | 46.7 | - | - | - | - | NS |
|  | concurrent |  | - | - | 55 | - | - | - | - |  |
|  | -XRT |  | 10.3 | - | - | - | - | - | - |  |
| Kobayashi 2018 | +XRT | 0 | 0 | 42.9 | 57.1 | - | - | - | - | RECIST 1.1 |
|  | -XRT | 25 | 0 | 40 | 55 | - | - | - | - |  |
| Hendriks 2019 | +XRT | 20.8 | - | 43.9 | - | - | - | 0.49 (0.31-0.79) | - | NS |
|  | -XRT |  | 27 |  | 60 | - | - |  | - |  |
| Zhang 2019 | +XRT | 25 | 25 | 53.1 | 50 | - | - | 0.97 (0.4-2.35) | 1.09 (0.41-2.92) | RECIST 1.1 |
|  | -XRT |  | 31.3 |  | 43.8 | - | - |  |  |  |
| Bjornhart 2019 | +XRT | 33.3 | 76.5 | - | 0 | - | - | - | - | RECIST 1.1 |
|  | -XRT |  | 0 | - | 0 | - | - | - | - |  |
| Goldberg 2020 | +XRT | 28.6 | 28.6 | - | 38 | - | - | - | 0.63 (0.30-1.30) | mRECIST 1.1 |
|  | -XRT |  | 23.8 | - |  | - | - | - |  |  |
| Checkmate012 2020 | -XRT | - | 8.3 | - | 16.7 | - | - | - | - | RECIST 1.1 |
| Ashinuma 2017 | -XRT | - | 25 | - | 50 | - | - | - | - | NS |
| Henon 2017 | - | 16.7 | 26.7 | - | 60 | - | - | - | - | NS |
| Molinier 2017 | - | - | 16.4 | - | 49 | - | - | - | - | RECIST 1.1 |
| Dumenil 2018 | +XRT | 0 | - | - | 14.3 | - | - | - | - | RECIST 1.1 |
|  | -XRT | 0 | - | - | 0 | - | - | - | - |  |
| Gauvain 2018 | sequential | 13 | 17.4 | 46.5 | 82.6 | - | - | - | - | RECIST 1.1 |
|  | concurrent | - | 0 |  | 100 | - | - | - | - |  |
|  | -XRT | 20 | 0 |  | 40 | - | - | - | - |  |
| Wakuda 2021 | +XRT | 69.2 | 76.9 | - | 84.6 | - | - | - | - | RECIST 1.1 |
|  | -XRT | 70 | 60 | - | 60 | - | - | - | - |  |
| Lucio 2019 | - | - | 16.6 | - | 40.1 | - | - | - | - | NS |
| Cortinovis 2019 | - | - | 18.9 | - | 49 | - | - | - | - | NS |
| Achim 2016 | - | - | - | - | - | - | - | - | 0.44 (0.31-0.94) | RECIST 1.1 |
| Ahmet 2021 | - | - | - | - | - | - | - | 0.45 (0.22-0.92) | 0.17 (0.04-0.76) | RECIST 1.1 |
| Lu 2021 | - | - | - | - | - | - | - | 0.61 (0.38-1.10) | 0.75 (0.30,1.30) | RECIST 1.1 |
| Goldman 2020 | - | - | - | - | - | - | - | - | 1.04 (0.62-1.76) | RECIST 1.1 |
| Borghaei 2015 | - | - | - | - | - | - | - | 0.80 (0.47-1.36) | 1.04 (0.62-1.76) | RECIST 1.1 |
| Martin 2019 | - | - | - | - | - | - | - | - | 0.73 (0.20-2.62) | RECIST 1.1 |
| Mansfield 2019 | - | - | 26.1 | - | - | - | - | 0.96 (0.73-1.25) | 1.04 (0.62-1.76) | RECIST 1.1 |
| Matthew 2019 | - | - | - | - | - | - | - | - | 0.64 (0.42-0.97) | NS |
| Muhammad 2018 | - | 80 | 80 | - | 80 | - | - | 0.67 (0.11-3.90) | - | RECIST 1.1 |
| Powell 2019 | - | - | 39 | - | - | - | - | 0.44 (0.31-0.62) | 0.48 (0.32-0.70) | RECIST 1.1 |
| Caicun 2020 | - | - | - | - | - | - | - | 0.14 (0.001-0.88) | - | RECIST 1.1 |
| Yunpeng 2020 | - | - | - | - | - | - | - | 0.58 (0.28-1.18) | - | RECIST 1.1 |
| Shepard 2019 | concurrent | - | 100 | - | 100 | - | - | 2.18 (0.72-6.62) | 0.99 (0.39-2.52) | NS |
|  | -XRT | - | 53.1 | - | - | - | - |  |  |  |
| Singh 2019 | +XRT | - | 88 | - | 96.8 | - | - | - | - | NS |
| Patruni 2019 | - | - | - | - | - | - | - | - | 0.77 (0.71-0.84) | RECIST 1.1 |
| Enright 2020 | concurrent | - | - | - | - | 39 | 96.9 | - | 0.46 (0.23-0.91) | RECIST 1.1 |
| Imber 2017 | concurrent | - | 60 | - | 91.1 | - | - | - | - | RECIST 1.1 |
|  | sequential | - | 40 | - | 88.9 | - | - | - | - |  |
| Srivastava 2018 | concurrent | 100 | 70 | - | - | - | 100 | - | - | RECIST 1.1 |
|  | sequential | 50 | 27.3 | - | - | - | 54.5 | - | - |  |
| Ahmed 2017 | concurrent | - | - | - | 53.8 | 0 | - | - | - | RECIST 1.1 |
|  | sequential | - | - | - | 0 | 57.1 | - | - | - |  |
| Gandhi 2018 | - | - | - | - | - | - | - | 0.42 (0.26-0.68) | 0.36 (0.20-0.62) | RECIST 1.1 |
| Schapira 2018 | concurrent | - | - | - | - | 37.5 | 100 | - | - | RECIST 1.1 |
|  | ICI after SRS | - | - | - | - | 66.7 | - | - | - |  |
|  | SRS after ICI | - | - | - | - | 100 | 80 | - | - |  |
| Wu 2019 | - | - | - | - | - | - | - | - | 0.82 (0.46-1.51) | RECIST 1.1 |
| Fehrenbacher 2018 | - | - | - | - | - | - | - | - | 0.59 (0.38-0.92) | RECIST 1.1 |
| Ernest 2021 | -XRT | 47.5 | 40 | 87.5 | 87.5 | - | - | - | - | RECIST 1.1 |
| David 2021 | - | 43.1 | 39.2 | - | 74.5 | - | - | 0.40 (0.25-0.64) | 0.43 (0.27-0.67) | RECIST 1.1 |
| Wang 2021 | - | 37.5 | - | - | - | - | - | - | - | RECIST 1.1 |
| Miranda 2021 | - | - | - | - | - | - | - | 0.53 (0.22-1.31) | 0.42 (0.14-1.26) | RECIST 1.1 |
| Caicun 2021 | - | - | - | - | - | - | - | 0.30 (0.15-0.60) | 0.45(0.20-0.85) | RECIST 1.1 |
| Natasha 2021 | - | - | - | - | - | - | - | 0.67 (0.35-1.30) | 0.58 (0.28-1.17) | RECIST 1.1 |

*Note. BM, brain metastases; mRECIST, modified response evaluation in solid tumours; HR, hazard ratio; PFS, progression-free survival; OS, overall survival; EORR, extracranial response rate; iORR,*

*intracerebral overall response; EDCR, extracranial disease control rate; iDCR, intracerebral disease control rate; RECIST, response evaluation criteria in solid tumours; ±XRT, recive/not recive radiotherapy .*
